# Successful Management of Coronavirus Disease 2019-Related Respiratory Failure Using High-Flow Nasal Cannula Therapy in a Patient with Underlying Pulmonary Artery Hypertension

**DOI:** 10.1155/2022/1774796

**Published:** 2022-11-09

**Authors:** Mariko Kotani, Tomoki Kohyama, Kiyoshi Moriyama, Tomoko Yorozu

**Affiliations:** Department of Anesthesiology, Kyorin University School of Medicine, 6-20-2 Shinkawa, Mitaka, Tokyo 181-8611, Japan

## Abstract

A case involving a 50-year-old woman (height, 155 cm; weight, 79.6 kg), who was undergoing home oxygen therapy (3.5 L/min), with an oxygen saturation (SpO_2_) of approximately 91% due to pulmonary artery hypertension (PAH) with mixed connective tissue disease, is reported. The patient developed coronavirus disease 2019- (COVID-19-) related respiratory failure, with an SpO_2_ of 78% on oxygen inhalation (3.5 L/min) and was admitted to the authors' hospital. In accordance with remdesivir, dexamethasone, and heparin treatment, high-flow nasal cannula (HFNC) therapy was selected to avoid intubation. At an initial HFNC setting of 70% oxygen with a flow rate of 50 L/min, SpO_2_ improved to 92% and her subjective symptoms improved. She was weaned from HFNC on day 5 of admission (day 14 of COVID-19 onset) and discharged home on day 14 of admission. In patients with PAH, the beneficial effects of HFNC to avoid endotracheal intubation were evident in avoiding hemodynamic instability and worsening respiratory failure.

## 1. Introduction

Among patients compromised by respiratory failure, those with pulmonary artery hypertension (PAH) are often difficult to manage because worsening respiratory status often causes circulatory fluctuations, which can lead to right ventricular failure and death. Herein, we report a case of coronavirus disease 2019- (COVID-19-) related respiratory failure in a patient with underlying PAH undergoing home oxygen therapy (HOT), who has successfully managed using high-flow nasal cannula (HFNC) therapy.

## 2. Case Presentation

A 50-year-old female (height, 155 cm; weight, 79.6 kg) undergoing HOT (3.5 L/min), with an oxygen saturation (SpO_2_) of approximately 91%, is reported. The patient was diagnosed with mixed connective tissue disease when she was 37 years of age and had been receiving prednisolone. During the course of her treatment, right heart catheterization revealed a mean pulmonary artery pressure of 27 mmHg, and she started receiving nifedipine, bosentan, sildenafil, and beraprost in accordance with HOT. She experienced worsening fever and cough for a few days and was referred to the authors' hospital because antibiotic therapy was ineffective.

**Figure 1:**
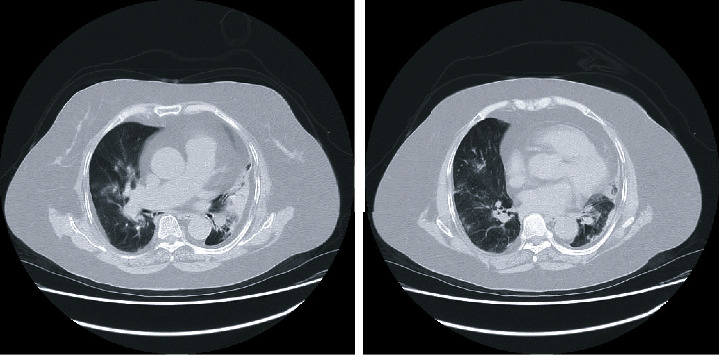
Chest computed tomography on admission to hospital. Scattered ground-glass opacities immediately below the right pleura and atelectasis in the left lower lung field are evident.

She was admitted to the hospital after testing positive for COVID-19 according to polymerase chain reaction testing. According to her clinical course, she was 10 days from the onset of COVID-19 and experienced severe respiratory failure, with a body temperature of 37.2°C, a heart rate of 103 beats/min, a blood pressure of 134/94 mmHg, and a respiratory rate of 30 breaths/min, with shoulder breathing and an SpO_2_ of 78% on oxygen inhalation (3.5 L/min). Chest computed tomography revealed scattered ground-glass opacities immediately below the right pleura and atelectasis in the left lower lung field (Figure 1).

Despite oxygen inhalation via reservoir mask (10 L/min), SpO_2_ increased only up to 83%. In typical cases of COVID-19-related respiratory failure, these parameters suggest that ventilator management by intubation is necessary. However, circulatory fluctuations and positive pressure ventilation associated with intubation were feared to adversely affect pulmonary hypertension. The patient also had a long history of smoking, was undergoing inhalation therapy for asthma, and was obese, with a body mass index of 33.1 kg/m^2^. Therefore, in accordance with remdesivir, dexamethasone, and heparin treatment, HFNC was selected to avoid intubation.

At an initial HFNC setting of 70% oxygen concentration and a flow rate of 50 L/min, SpO_2_ improved to 92%, and her subjective symptoms also improved. At this time point with 70% of oxygen inhalation, her arterial blood gas showed a pH of 7.419, PaCO_2_ of 37.3 mmHg, PaO_2_ of 72.5 mmHg, and bicarbonate of 23.7 mmol/L. Her PaO_2_/F_I_O_2_ ratio of 104 indicated moderate hypoxic respiratory failure. Her respiratory rate increased to >30 breaths/min on day 1 of admission. However, her respiratory rate decreased the next day, in addition to oxygen concentration. Her ROX (i.e., ratio of oxygen saturation as measured by pulse oximetry/fraction of inspired oxygen to respiratory rate) indices were 4.28 at 2 h following initiation of HFNC and decreased to 3.90 at 6 h; however, it increased to 6.67 at 12 h. She continued to progress without major complications. Settings of HFNC were changed while keeping SpO_2_ around 86% to 92% as follows: 60% of oxygen at 50 L/min of flow (day 1), 50% of oxygen at 50 L/min of flow (day 2), 40% of oxygen at 40 L/min of flow (day 3), and 35% of oxygen at 40 L/min of flow (day 4). After applying 30% of oxygen at 40 L/min of flow, the patient was weaned from HFNC on day 5 of admission (day 14 of COVID-19 onset). The patient was discharged on day 14 of admission.

## 3. Discussion

Patients with underlying chronic health conditions are at increased risk for developing severe COVID-19 [1]. In patients with PAH, elevated pulmonary artery pressure may lead to right ventricular failure and death, with high mortality during hospitalization, both due to PAH-related causes and noncardiac conditions [2] [3]. Sulica et al. [4] reported a remarkably high COVID-related mortality rate of 36.36% at the beginning of the COVID-19 pandemic in New York City, in the United States. In our patient, respiratory failure due to SARS-CoV-2 infection led to hypoxemia, and oxygen therapy via masks was ineffective in increasing oxygenation, and ventilator management by intubation appeared to be necessary. However, in patients with PAH, intubation should be avoided if possible, because positive pressure ventilation increases intrathoracic pressure impeding preload in the right ventricle, and sedatives affect cardiac function and cause vasodilation, leading to systemic hypotension and hemodynamic collapse [1]. Therefore, HFNC was selected.

To avoid the adverse effects of mechanical ventilation, noninvasive ventilation (NIV) or HFNC, which was selected in this case, can be used. We previously reported [5] a patient with reperfusion pulmonary edema following percutaneous transluminal pulmonary angioplasty, who was successfully managed using HFNC. Due to the simplicity of the technique, lower equipment costs, and remarkable patient tolerance to treatment, HFNC is sometimes superior to NIV in patients with acute respiratory failure.

The issue of whether to use HFNC or NIV for COVID-19-related respiratory failure has been controversial [6], and variability exists among the current guidelines. Although both HFNC and NIV are useful in avoiding mechanical ventilation, concerns remain that delayed tracheal intubation may lead to worse outcomes. Because the adverse effects of mechanical ventilation are more evident in patients with PAH than in those without PAH, the beneficial effects of HFNC to avoid tracheal intubation are more justifiable.

Aerosolized transmission from patients to treating healthcare professionals remains a concern, and the use of negative pressure wards and adequate personal protective equipment for all medical staff has helped to reduce anxiety. In the early stages of the current COVID-19 pandemic, initial concerns about the risk of bioaerosol dispersion and delayed intubation led some scientific societies to limit or not recommend the application of HFNC and other noninvasive respiratory support devices with different and sometimes opposite recommendations between national and international organizations [7]. In this case, avoiding endotracheal intubation helped reduce the necessity of medical staff for patient care and helped reduce anxiety among medical personnel. Because no medical staff member got COVID-19 infection while treating this patient, further applications of HFNC to COVID-19 patients were enhanced in our intensive care unit [8].

## 4. Conclusion

We encountered a case of COVID-19-related respiratory failure in a patient with PAH undergoing HOT. In patients with PAH, the beneficial effects of HFNC to avoid endotracheal intubation were evident in avoiding hemodynamic instability and worsening respiratory failure from mechanical ventilation.

## Data Availability

Readers can access the data through the corresponding author via e-mail.
